# Supplementary material for: Apical dehydration impairs the cystic fibrosis airway epithelium barrier via a β1-integrin/YAP1 pathway
Source: Life Sci Alliance. 2024 Feb 9;7(4):e202302449. doi: 10.26508/lsa.202302449 (PMC10858171; doi:10.26508/lsa.202302449)
Supplement: Supplementary file 10 [file LSA-2023-02449_SdataFS3.2.pdf]

<u>Figure S3C</u>

YAP1 and GAPDH

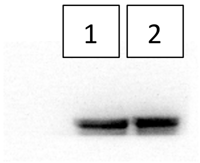

<u>YAP1 (80kDa): lanes 1 and 2</u>.

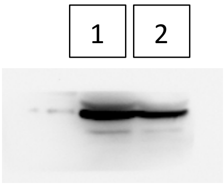

<u>GAPDH (37kDa): lanes 1 and 2</u>.

<u>Figure S3E</u>

pYAP397, YAP1 and GAPDH

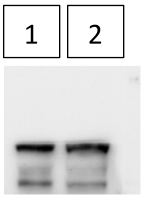

<u>pYAP397 (80kDa): lanes 1 and 2</u>.

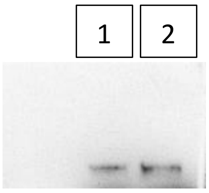

<u>YAP1 (80kDa): lanes 1 and 2</u>.

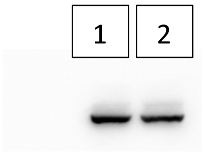

<u>GAPDH (37kDa): lanes 1 and 2</u>.
