# Supplementary figures and images for: Apical dehydration impairs the cystic fibrosis airway epithelium barrier via a β1-integrin/YAP1 pathway
Source: Life Sci Alliance. 2024 Feb 9;7(4):e202302449. doi: 10.26508/lsa.202302449 (PMC10858171; doi:10.26508/lsa.202302449)

**Figure S4A**

YAP1 and  $\beta$ -actin

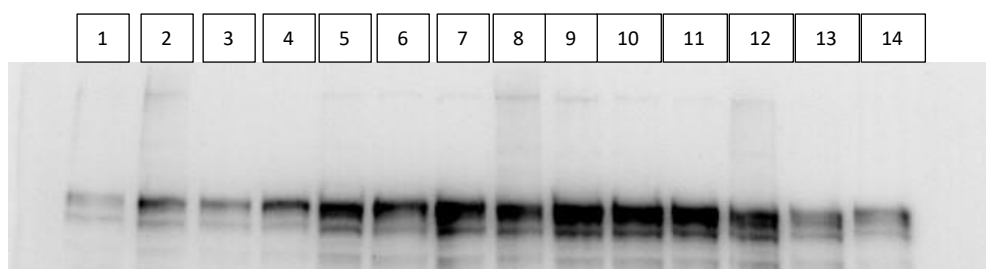

YAP1 (80kDa): lanes 1 to 14.

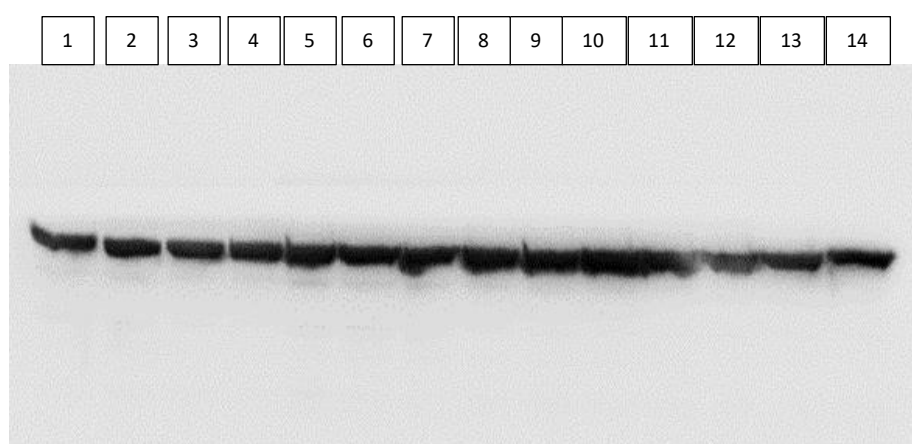

$\beta$ -actin (42kDa): lanes 1 to 14.

Supplement: Supplementary file 11 [file LSA-2023-02449_SdataFS4.1.pdf]

**Figure S5C**

TAZ and GAPDH

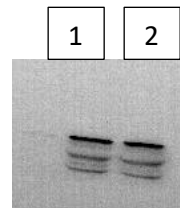

TAZ (44kDa): lanes 1 and 2.

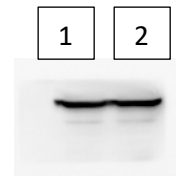

GAPDH (37kDa): lanes 1 and 2.

Supplement: Supplementary file 16 [file LSA-2023-02449_SdataFS5.2.pdf]
