# Supplementary material for: Apical dehydration impairs the cystic fibrosis airway epithelium barrier via a β1-integrin/YAP1 pathway
Source: Life Sci Alliance. 2024 Feb 9;7(4):e202302449. doi: 10.26508/lsa.202302449 (PMC10858171; doi:10.26508/lsa.202302449)
Supplement: Supplementary file 13 [file LSA-2023-02449_SdataF6.1.pdf]

Figure 6A

CFTR-CTL: YAP1 and GAPDH
CFTR-KD: YAP1 and GAPDH

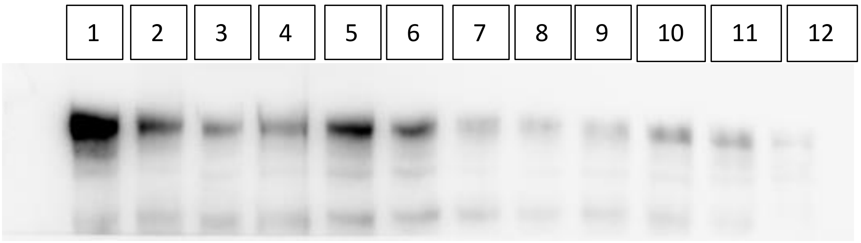

YAP1 (80kDa), CFTR-CTL samples: lanes 1 to 6. Other lanes correspond to conditions not used for the article.

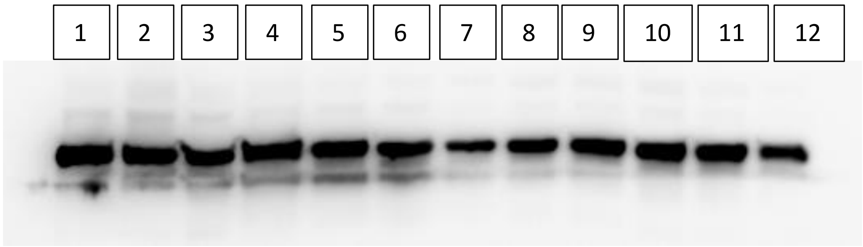

GAPDH (37kDa), CFTR-CTL samples: lanes 1 to 6. Other lanes correspond to conditions not used for the article.

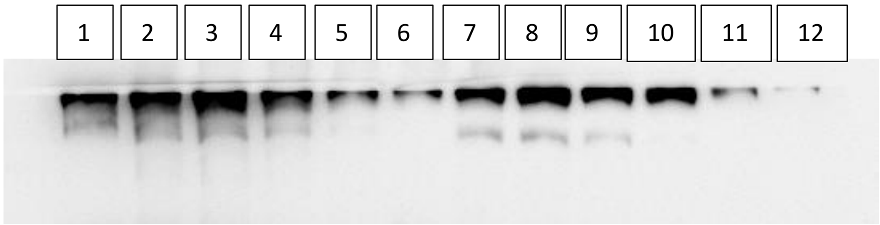

YAP1 (80kDa), CFTR-KD samples: lanes 7 to 12. Other lanes correspond to conditions not used for the article.

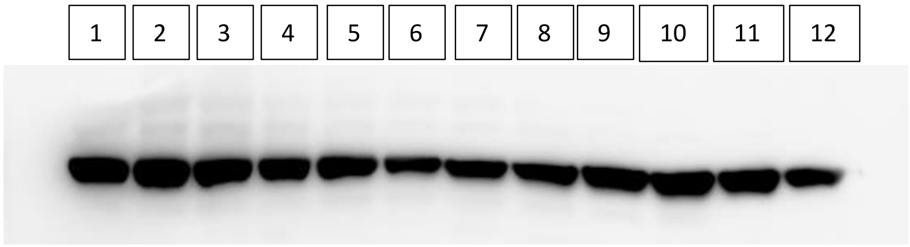

GAPDH (37kDa), CFTR-KD samples: lanes 7 to 12. Other lanes correspond to conditions not used for the article.

Figure 6C

pYAP397, YAP1 and β-actin

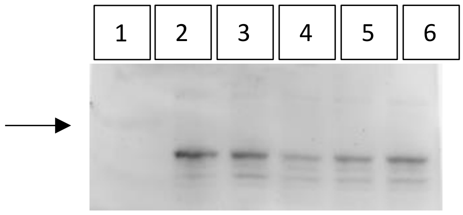

pYAP397 (80kDa): lanes 4 to 6. Lane 1: molecular weight ladder. Arrow at 100kDa. Other lanes correspond to conditions not used for the article.

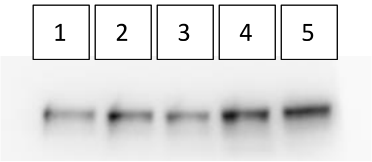

YAP1 (80kDa): lanes 3 to 5. Other lanes correspond to conditions not used for the article.

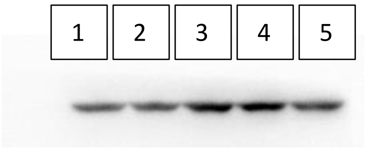

β-actin (42kDa): lanes 3 to 5. Other lanes correspond to conditions not used for the article.
