# Supplementary material for: Apical dehydration impairs the cystic fibrosis airway epithelium barrier via a β1-integrin/YAP1 pathway
Source: Life Sci Alliance. 2024 Feb 9;7(4):e202302449. doi: 10.26508/lsa.202302449 (PMC10858171; doi:10.26508/lsa.202302449)
Supplement: Supplementary file 19 [file LSA-2023-02449_SdataF7.1.pdf]

Figure 7A

YAP1 and β-actin

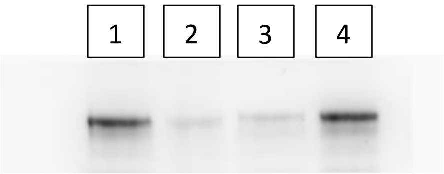

YAP1 (80kDa): lanes 1 and 2. Other lanes correspond to conditions not used for the article.

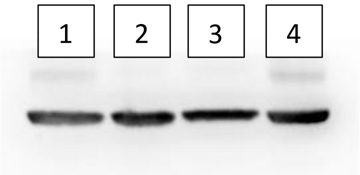

β-actin (42kDa): lanes 1 and 2. Other lanes correspond to conditions not used for the article.

Figure 7B

E-cadherin and β-actin

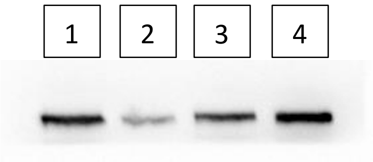

E-cadherin (135kDa): lanes 1 and 2. Other lanes correspond to conditions not used for the article.

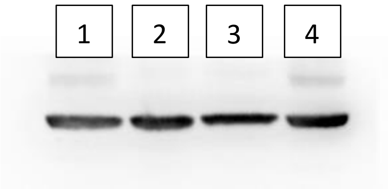

β-actin (42kDa): lanes 1 and 2. Other lanes correspond to conditions not used for the article.

Figure 7C on top

Claudin-3 and β-actin

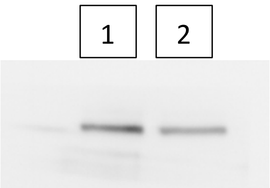

Claudin-3 (18kDa): lanes 1 and 2.

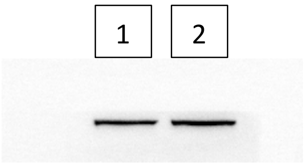

β-actin (42kDa): lanes 1 and 2.

Figure 7C at the bottom

Claudin-2 and β-actin

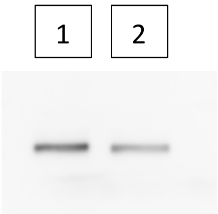

Claudin-2 (25kDa): lanes 1 and 2.

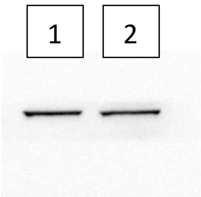

β-actin (42kDa): lanes 1 and 2.

Figure 7D on top

β-catenin and β-actin

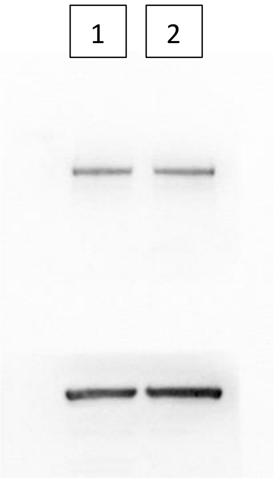

β-catenin (88kDa): lanes 1 and 2 on top and β-actin (42kDa): lanes 1 and 2 below.

Figure 7D at the bottom

α1-catenin and β-actin

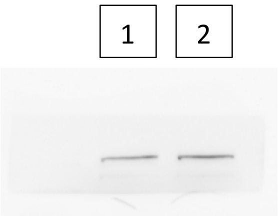

α1-catenin (100kDa): lanes 1 and 2.

1 2

β-actin (42kDa): lanes 1 and 2.
