# Supplementary material for: Apical dehydration impairs the cystic fibrosis airway epithelium barrier via a β1-integrin/YAP1 pathway
Source: Life Sci Alliance. 2024 Feb 9;7(4):e202302449. doi: 10.26508/lsa.202302449 (PMC10858171; doi:10.26508/lsa.202302449)
Supplement: Supplementary file 21 [file LSA-2023-02449_SdataFS7.pdf]

Figure S7A

YAP1 and β-actin

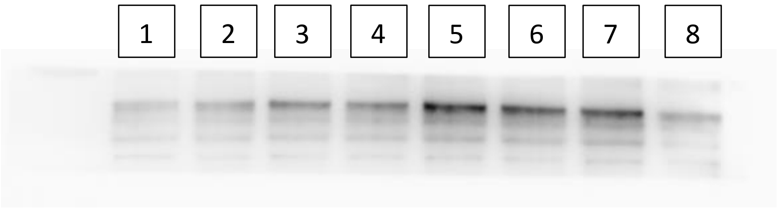

YAP1 (80kDa): lanes 1 to 8.

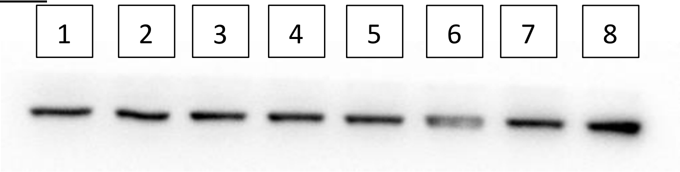

β-actin (42kDa): lanes 1 to 8.

Figure S7B claudin-3

Claudin-3 and β-actin

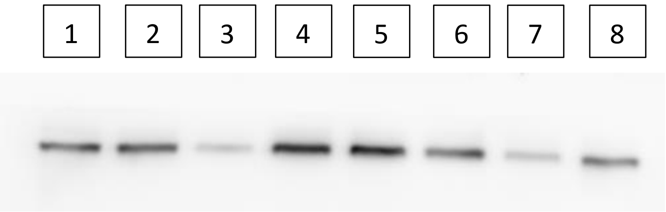

Claudin-3 (18kDa): lanes 1 to 8.

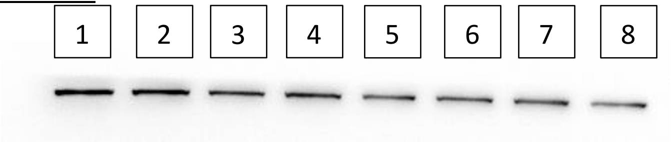

β-actin (42kDa): lanes 1 to 8.

Figure S7B claudin-2

Claudin-2 and β-actin

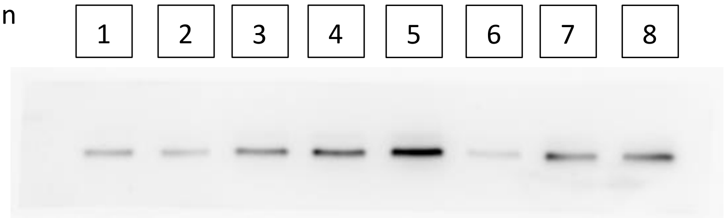

Claudin-2 (25kDa): lanes 1 to 8.

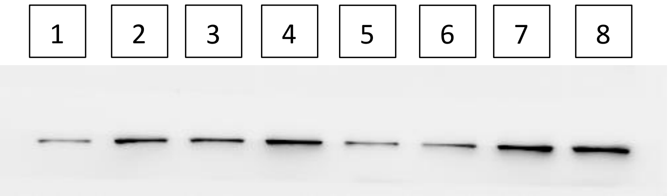

β-actin (42kDa): lanes 1 to 8.

Figure S7B E-cadherin

E-cadherin and β-actin

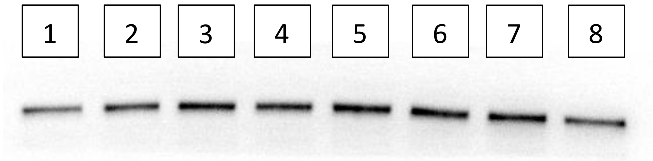

E-cadherin (135kDa): lanes 1 to 8.

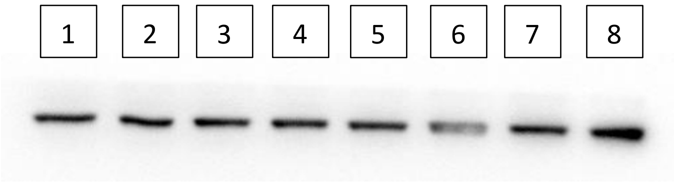

β-actin (42kDa): lanes 1 to 8.

Figure S7B β-catenin

β-catenin and β-actin

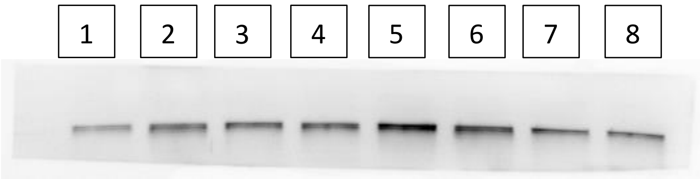

β-catenin (88kDa): lanes 1 to 8.

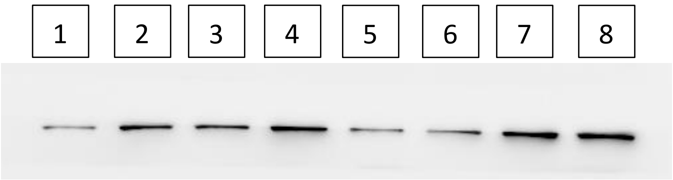

β-actin (42kDa): lanes 1 to 8.

Figure S7B α1-catenin

α1-catenin and β-actin

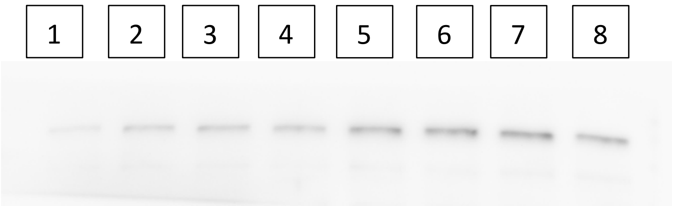

α1-catenin (100kDa): lanes 1 to 8.

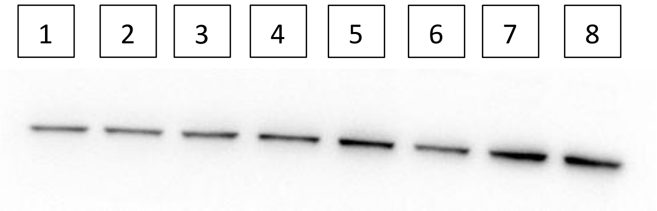

β-actin (42kDa): lanes 1 to 8.
