# Supplementary material for: Apical dehydration impairs the cystic fibrosis airway epithelium barrier via a β1-integrin/YAP1 pathway
Source: Life Sci Alliance. 2024 Feb 9;7(4):e202302449. doi: 10.26508/lsa.202302449 (PMC10858171; doi:10.26508/lsa.202302449)
Supplement: Supplementary file 22 [file LSA-2023-02449_SdataFS8.1.pdf]

Figure S8A

Total β1-integrin and β-actin

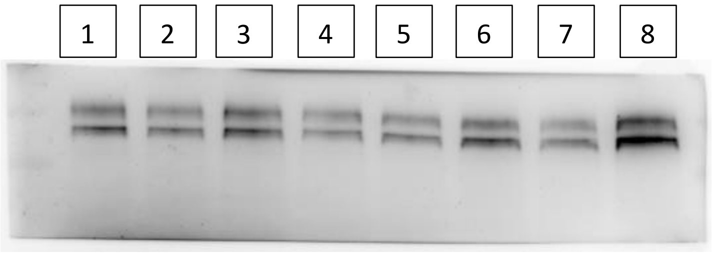

Total β1-integrin (110-130kDa): lanes 1 to 8.

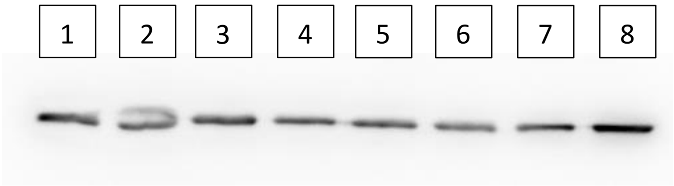

β-actin (42kDa): lanes 1 to 8.
