# Supplementary material for: Apical dehydration impairs the cystic fibrosis airway epithelium barrier via a β1-integrin/YAP1 pathway
Source: Life Sci Alliance. 2024 Feb 9;7(4):e202302449. doi: 10.26508/lsa.202302449 (PMC10858171; doi:10.26508/lsa.202302449)
Supplement: Supplementary file 24 [file LSA-2023-02449_SdataF8.1.pdf]

Figure 8B

YAP1 and β-actin

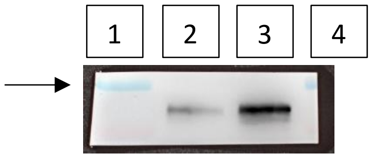

YAP1 (80kDa): lanes 2 and 3. Lanes 1 and 4: molecular weight ladder. Arrow at 100kDa.

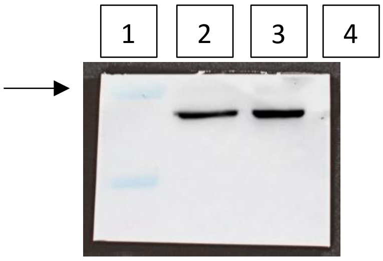

β-actin (42kDa): lanes 2 and 3. Lanes 1 and 4: molecular weight ladder. Arrow at 55kDa.
