# Supplementary material for: Apical dehydration impairs the cystic fibrosis airway epithelium barrier via a β1-integrin/YAP1 pathway
Source: Life Sci Alliance. 2024 Feb 9;7(4):e202302449. doi: 10.26508/lsa.202302449 (PMC10858171; doi:10.26508/lsa.202302449)
Supplement: Supplementary file 26 [file LSA-2023-02449_TableS1.docx]

**Supplementary Table 1.** Key reagents and resources used in this study.

| **REAGENT or RESOURCE** | **SOURCE** | **IDENTIFIER** |
| --- | --- | --- |
| Calu-3 cell line | ATCC | HTB-55™ |
| Custom lentiviral particles: Lentiviral production of custom clone, cloned into pLKO-puro-IPTG-3xLacO vector | Merck | CSTVRS |
| Clone sequence: CCCAGTTAAATGTTCACCAAT | Merck | TRCN0000107265 |
| Hexadimethrine bromide | Merck | H9268 |
| Isopropyl β-D-1-thiogalactopyranoside | Merck | I6758 |
| Puromycin | InvivoGen | QLL-42-01 |
| **Antibodies** | | |
| YAP1 | Cell Signaling | 14074S |
| pYAP397 | Cell Signaling | 13619S |
| TAZ | Abcam | ab84927 |
| total β1-integrin | Cell Signaling | 4706S |
| CD29 Clone 9EG7 | BD Pharmingen, BD Biosciences | 550531 |
| Claudin-3 | Abcam | ab15102 |
| Claudin-2 | Abcam | ab53032 |
| GAPDH | Merck | MAB374 |
| β-actin | Sigma | A1978 |
| α1-catenin | Abcam | ab51032 |
| β-catenin | Cell Signaling | 8480S |
| E-cadherin | Cell Signaling | 3195S |
| Goat anti-Rabbit HRP | Sigma | Cat# A8275, RRID:AB_258382 |
| Goat anti-Mouse HRP | Sigma | Cat# A5278, RRID:AB_258232 |
| Alexa Fluor 647 goat anti-rabbit (H+L) | ThermoFisher | Cat# A-21245, RRID:AB_2535813 |
| Alexa Fluor™ 647 goat anti-mouse (H+L) | ThermoFisher | A21236 |
| Alexa Fluor 568 goat anti-rat | Invitrogen | A11077 |
| Alexa Fluor™ 568 goat anti-rabbit (H+L) | ThermoFisher | A11011 |
| Alexa Fluor™ 568 goat anti-mouse (H+L) | ThermoFisher | A11031 |
| **Other reagents** | | |
| MEM GlutaMAX | Gibco® | 41090-28 |
| Fetal Bovine Serum (FBS) | Gibco® | 10270-106 |
| Non-essential amino acids 100X | Gibco® | 11140-035 |
| HEPES 1M | Gibco® | 15630-056 |
| Sodium pyruvate 100X | Gibco® | 11360-039 |
| Penicillin/Streptomycin/Fungizone® | BioConcept | 4-02F00-H |
| Nonidet-P40 | AppliChem | A1694 |
| complete™ Protease Inhibitor Cocktail | Roche | 04693124001 |
| Pierce BCA protein assay kit | ThermoFisher | 23228 |
| SDS-PAGE | Bio-Rad | 161-0301 |
| PageRuler Plus Prestained Protein Ladder | ThermoScientific | 26619 |
| SuperSignal West Pico PLUS Chemiluminescent Substrate | ThermoScientific | 34580 |
| Porablot NCP nitrocellulose membrane | Macherey-Nagel | 741280 |
| BSA | Sigma | A7906 |
| PBS | Gibco® | 14190-094 |
| Tween | PanReac Applichem | A4974 |
| HRP substrate Immobilon™ Western | Millipore | WBKLS0500 |
| Trypsin-EDTA 10X | Gibco® | 15400-054 |
| Paraformaldehyde (PFA) | Sigma | 158127 |
| Triton 100X | Sigma | T8787 |
| Superblock | ThermoScientific | 37580 |
| DAPI | AppliChem | A4099 |
| Phalloidin-iFluor 647 | Abcam | ab176759 |
| Vectashield mounting medium | Reactolab | H-1000 |
| RNeasy kit | Qiagen | 74106 |
| QuantiTect Reverse Transcription Kit | Qiagen | 205311 |
| PowerUp™ SYBR™ Green Master Mix | Appliedbiosystems | A2574 |
| Flipper-TR probe | Spirochrome | SC020 |
| Na acetate | Sigma | S2889 |
| Tris | Sigma | T1503 |
| Dextran Tetramethylrhodamine 10000 MW | Molecular Probes | D1868 |
| Cycloheximide | Sigma | 01810 |
| Actinomycin D | Merck | A9415 |
| Amitriptyline | Sigma | A8404 |
