# Supplementary material for: Apical dehydration impairs the cystic fibrosis airway epithelium barrier via a β1-integrin/YAP1 pathway
Source: Life Sci Alliance. 2024 Feb 9;7(4):e202302449. doi: 10.26508/lsa.202302449 (PMC10858171; doi:10.26508/lsa.202302449)
Supplement: Supplementary file 27 [file LSA-2023-02449_TableS2.docx]

| Gene | Forward | Reverse |
| --- | --- | --- |
| YAP1 | CCTTCTTCAAGCCGCCGGAG | CAGTGTCCCAGGAGAAACAGC |
| TAZ | GATCCTGCCGGAGTCTTTCTT | CACGTCGTAGGACTGCTGG |
| 18S | GTAACCCGTTGAACCCCATT | CCATCCAATCGGTAGTAGCG |
| GAPDH | TGGTATCGTGGAAGGACTCATGAC | ATGCCAGTGACGTTCCCGTTCAGC |

**Supplementary Table 2.** List of primer pairs used for qPCR.
